# Diving Medicine: An Exciting Journey Through Time and Future Prospects

**DOI:** 10.7759/cureus.56947

**Published:** 2024-03-26

**Authors:** Dimitra Ioanna Lampropoulou, Dimitrios Papageorgiou, Evangelia Pliakou

**Affiliations:** 1 Clinical Research, Econcare, Athens, GRC; 2 Obstetrics and Gynecology, Athens Naval Hospital (NNA), Athens, GRC; 3 Oncology, Athens Naval Hospital (NNA), Athens, GRC

**Keywords:** hyperbaric oxygen treatment, hyperbaric medicine, history, diving medicine, decompression sickness, decompression illness

## Abstract

Humans, led by their eternal wish to explore the unknown, have always wanted to perfect their diving skills and conquer the sea world. The adverse conditions experienced by divers brought about medical problems and a new field of medicine. Diving medicine serves the identification, treatment, and precautions against illnesses that are related to diving activities. While the development of diving equipment is advancing, divers have had the chance to reach greater depths for a longer time. Along with this success, a novel medical condition under the term ‘decompression illness’ (DCI) was introduced. Although the history of hyperbaric medicine is very long, progress in the field of mechanics has offered great contributions to the management of the disease. The first attempt at DCI guidelines was made by the US Navy in 1944-1945 and resulted in the creation of hyperbaric treatment tables. These tools received international recognition, offering a major advance. Hyperbaric-Diving Medicine holds an important place in modern medical science nowadays with indications for various diseases. At the same time, there is great scientific interest and a lot of research in the use of hyperbaric oxygen for several medical disorders, demonstrating great potential.

## Introduction and background

Humans, led by their eternal wish to explore the unknown and extend their knowledge and understanding of the world, have always wanted to perfect their diving skills. The conquest of the sea world, as early as the 4th century BC, has served not only to obtain goods and materials but also to develop marine trade and military operations. However, during diving activities, people face adverse working conditions, very different from what their bodies are genetically structured to sustain; these conditions have brought about a novel kind of medical problems that extend from barotrauma of the inner ear, first described by Aristotle, to decompression illness (DCI), which was identified much later [1]. Diving medicine, as its name indicates, serves in the identification, treatment, and precautions against illnesses related to diving activities. Its development is equivalent to the progress and discoveries made in the field of diving equipment and technology. An important milestone in the history of diving medicine was the development of hyperbaric medicine at the beginning of the 20th century; this is primarily applied in the treatment of DCI, known otherwise as the Divers’ Disease. Hyperbaric medicine, as defined by the Undersea and Hyperbaric Medical Society (UHMS), is the science that involves the medical use of 100% oxygen inside a decompression chamber, where the ambient pressure is greater than sea-level atmospheric pressure [2, 3]. Therefore, the use of the two terms, diving and hyperbaric medicine, are considered rather synonymous. This review aims to present the most important and interesting milestones in the history of diving medicine, which extends over 2400 years and is inseparable from the history of human evolution and culture. These milestones, extending from the 16th century and beyond, describe the establishment of the foundations which govern modern diving medicine and are geographically limited to Europe and America.

## Review

An electronic search in scientific databases (PubMed, Google Scholar, Scopus) was conducted during October 2023 to January 2024. The most relevant articles were retrieved using the following keywords: "hyperbaric oxygen treatment", "hyperbaric medicine", "history", "diving medicine", "decompression sickness", "decompression illness". Further screening excluded irrelevant or duplicated articles, and the content of the remaining publications was evaluated for its relevance to the current review by all authors.

Brief history of diving

The first divers, dating back to 4500 BC, were sponge divers or pearl fishermen who could dive up to a depth of 30 meters without using any equipment besides auxiliary weights to reach the seabed faster. In Homer’s Iliad (700 BC), several military diving operations are mentioned during the Trojan War, whereas Herodotus mentions that the famous diver Scyllias (400 BC) was hired by Xerxes to search for treasures in the sunken Persian ships [4]. About a century later, in 300 BC, Aristotle described in detail the perforation of the tympanic membrane in divers. Furthermore, Alexander the Great introduced the first diving equipment, a barrel made of glass, to submerge in the area of the Bosporus in 320 BC. Since then, many efforts have been made to enable divers to extend their duration underwater. Some of the milestones in the History of Diving and Diving Medicine are mentioned in Table 1.

**Table 1:** Some important milestones in the history of diving and diving medicine from the 16th century onward.

Year	Milestones in the History of Diving Medicine	Reference
1500	The first diving and underwater devices were designed by Leonardo Da Vinci.	[4]
1620	The precursor of submarine devices, a diving bell operating with 1 atmosphere (atm) pressure, was built by Cornelius Drebbel.	[4]
1670	Boyle described the decompression phenomenon for the first time (“bubble in the eye of a snake in a vacuum”).	[1, 4]
1680	The precursor of the contemporary SCUBA devices and flippers was designed by Giovanni Borelli.	[5]
1692	Edmund Halley created a diving bell with a surface air supply system.	[4]
1774	Freminet used a diving apparatus with a continuous air supply to stay at a depth of 55 feet for one hour.	[4]
1841	The first description of decompression sickness symptoms in humans was made in 1841 by Triger in miners working in elevated pressure galleries.	[1]
1854	Pol and Watelle of France observed that recompression relieved the symptoms of decompression sickness.	[1, 4]
1878	Bert described the dissolution of nitrogen in tissues and blood and the formation of bubbles. Also, he found that oxygen became toxic at an elevated pressure (more than 33 ft), and this toxic effect manifested as muscle convulsions (the “P. Bert Phenomenon”).	[1]
1920	A mixture of gases, mainly helium (He), and oxygen (O2), was used for diving; diving depth extended to 200 m.	[4]
1943	Jacque-Yves Cousteau and Emile Gagnan invented the Aqua-Lung, the diving regulator used for the first Scuba equipment	[4]

DCI and other diving disorders

While the development of diving equipment was advancing, divers had the chance to reach increasingly greater depths for longer periods of time. Along with this success, a novel medical condition known as DCI was introduced. DCI encompasses two diseases: decompression sickness (DCS) and arterial gas embolism (AGE).

Pathogenesis

DCI develops due to the presence of bubbles of an inactive gas that has dissolved in the body tissues during diving. These bubbles are released while the diver is ascending. This event is attributed to the conversion of dissolved gas in blood and body tissues back into its gaseous state. During diving, the total amount of dissolved gas in a liquid is directly proportional to its partial pressure above it (Henry’s Law) [6]. The amount of nitrogen dissolved in the blood and body tissues during diving depends on i) the depth at which the dive takes place and ii) the duration of the body's exposure to that depth and pressure. Furthermore, the rate and amount of nitrogen released in tissues depend on i) tissue blood flow, ii) gas diffusion, and iii) gas solubility. During ascent, the total pressure of the dissolved gases exceeds the total pressure of the inhaled gases, due to the fact that ascending causes a decrease in ambient pressure, resulting in supersaturation. Tissue gas desaturation during diving leads to the formation of bubbles in the cardiovascular system and tissues [7].

The formation of bubbles is the first in a cascade of phenomena and, depending on their quantity and location, may generate symptoms [7,8]. In symptomatic cases, gas bubbles in human tissues can cause: Mechanical effects, such as distortion or damage to human tissues due to direct pressure, and arterial obstruction resulting in ischemia; Non-mechanical effects, such as activation of leukocytes, platelets, the coagulation and complement cascades, peripheral vascular obstruction along with microcirculation disorders, and an increase in capillary permeability which may lead to edema formation. The symptoms are usually classified as Type I and II and manifest 15 minutes to 36 hours after the dive; in most cases, they occur within six hours. Moreover, tissues with a high-fat content, (e.g., the brain or spinal cord), are particularly susceptible, given that nitrogen dissolves very readily in fats [4, 5, 9].

*Type I Symptoms* Musculoskeletal form (often called 'the bends'): The most common symptom (90% of cases) is described as soreness or pain around or close to the joints, ranging from niggle to unbearable. The upper limbs of divers are affected more often (3:1) in comparison to the lower limbs. Joints that are usually affected include the shoulders, elbows, hips, knees, and the lower parts of the arm. This is attributed to bubble formation near the tendons and ligaments around the joints. Symptoms from the lymphatic system: These manifest as pain and swelling of particular groups of lymph nodes along with soft tissue edema. Skin bends: These manifest as itching with or without an exfoliating rash, or a very distinctive rash, also known as marbling (livedo reticularis), resembling erythema. It usually appears on the back and chest, followed by linear, reddish imprints. *Type II Symptoms* Central nervous system: The symptoms include headache (tension headache or migraine), excessive fatigue, confusion and disorientation, cognitive dysfunction, visual disturbances, muscular weakness (usually unilateral), hemiparesis, hemiplegia, or loss of consciousness, which may lead to shock or even death [10].

Spinal cord: The patient may report sensory disorders in the lower limbs, progressive instability, and loss of sphincter control. Instability is associated with muscular weakness that develops into paraparesis or paraplegia. Moreover, pain in the lumbar or back may be present, before and/or concurrently with the onset of symptoms [10]. *Pulmonary Disease (Chokes)* The patient often reports an inspiratory burning sensation and substernal discomfort of increasing intensity. This symptom is initially experienced during coughing and later during both inhalation and expiration. It can also be accompanied by dyspnea, respiratory distress, and non-productive coughing, which may constantly deteriorate and lead to severe respiratory distress. *Inner Ear Disease (Staggers)* Symptoms such as tinnitus, hearing loss, rotational vertigo, nausea, vomiting, nystagmus, and walking disorders are also associated with DCI. *Shock* The diver appears to have lost consciousness and becomes confused and hemodynamically unstable. These symptoms are attributed to i) loss of vascular tone, ii) myocardial suppression, iii) hypoxia and acidosis, iv) pulmonary embolism, and v) severe hemoconcentration; urgent assessment and interventions are required [11].

Finally, the “subclinical decompression disease” type has also been described. This entity is defined as a medical condition in which the patient does not report any specific symptoms. Instead, mild symptomatology is reported, such as fatigue, a "heavy head," and mild aching joints. This type is probably attributed to a number of "silent" bubbles and is more often linked with deep dives (>40 m) using air as a respiratory means. About 95% of cases develop mild symptoms within the first 3 hours after being removed from the elevated pressure environment.

The main clinical manifestations and their symptoms are summarized in Table 2. 

**Table 2:** Decompression illness (DCI) includes two clinical entities, decompression sickness (DCS) and arterial gas embolism (AGE), which are the most common and significant problems related to diving. The main symptoms of DCI are detailed in the table above.

Decompression illness	Clinical manifestation	Comments
Subclinical Decompression Disease	Absence of, or mild symptomatology: fatigue, "heavy head", mild aching joints.	Usually self-limiting.
DCI- Type I Symptoms (Less Severe)	Musculoskeletal form (90%): pain and swelling of lymph nodes or soft tissue; skin bends.	Upper limbs are affected more often (3:1) in comparison to the lower limbs; itching with or without an exfoliating rash, or marbling (livedo reticularis), usually occurs on the back and chest, followed by linear, reddish imprints.
DCI- Type II Symptoms (More Severe)	Excessive fatigue, muscular weakness; visual disturbances; CNS-spinal cord: headache (tension headache or migraine), confusion and disorientation, cognitive dysfunction, hemiparesis – hemiplegia, loss of consciousness; shock, death.	Symptoms often mimic spinal cord trauma: more often unilateral muscular weakness, sensory disorders, especially in the lower limbs, progressive instability that could develop into paraparesis or paraplegia, and loss of sphincter control. Pain in the lumbar or back may exist, known as pelvic girdle pain, before and/or concurrently with the onset of symptoms (about 30%).
Inner Ear Disease (Staggers)	Tinnitus, hearing loss, rotational vertigo, nausea, vomiting, nystagmus, and walking disorders.	
Pulmonary Disease (Chokes)	Coughing, dyspnoea, and respiratory distress.	Inspiratory burning sensation, non-productive coughing, and substernal discomfort of increasing intensity; severe respiratory distress which could lead to death.
Pulmonary Barotrauma and Arterial Gas Embolism (AGE)	Pneumothorax; mediastinal emphysema; myocardial infarction or arrhythmias (coronary artery embolization); dizziness, headache, and profound anxiousness; unresponsiveness; ischemic stroke or seizures (cerebral artery embolization); shock; death.	Gas embolization occurs when a rupture into the pulmonary veins allows alveolar gas to enter systemic circulation. Gas bubbles expand as one ascends, increasing the severity of symptoms. Differentiating cerebral AGE from Type II DCI is based upon the onset of symptoms (typically occurring within 10-20 minutes after surfacing) and the involvement of multiple systems.
Shock	Tachycardia; hypotension; thromboembolic events; death.	Hemodynamic instability requires urgent management.

History of the disease

The first study investigating the rationale of decompression disease was carried out by Sir Robert Boyle when he tried to induce the symptoms related to this disease in a snake placed inside a vacuum pump. Boyle concluded that the sudden drop in pressure was more likely to cause bubble formation in the tissues of the snake’s body [12]. The first description of decompression sickness symptoms in humans was made in 1841 by Triger in miners working in elevated pressure galleries (to prevent water from entering). Triger noticed that some miners, after leaving the galleries, experienced cramps and myalgia that receded after drinking alcohol and rubbing the painful body area [1]. Some years later, in 1854, Pol and Watelle began to observe the phenomenon of decompression illness. They noticed it most frequently appeared when someone was leaving an environment of increased pressure ("one pays only on leaving”). Returning to the increased pressure site contributed to the amelioration of the symptoms [2]. However, the first scientific approach to the problem was made by the French scientist Paul Bert in 1878. Bert concluded that breathing air under high pressure led to the dissolution of nitrogen in tissues and blood. Afterwards, as the pressure dropped rapidly, the nitrogen formed bubbles (due to returning to its gaseous state). Bert also found that oxygen became toxic at elevated pressures (more than 33 ft), manifesting as muscle convulsions (the “P. Bert Phenomenon”). Following these conclusions, divers and workers in elevated pressure environments began to apply re-pressurization procedures to treat symptoms of the disease. Until then, divers either limited the depth of their dives or suffered from the consequences of the disease [13]. The term "Bends" was introduced as a synonym for decompression disease during the construction of the Brooklyn Bridge, originating from the typical rocking walk of the women of that time, called the "Grecian Bend". The distinct walk that the workers exhibited during the construction of the bridge due to DCI was also called the “Grecian Bend,” which gradually evolved into "Bends” [14]. The Greek professor Michael Katsaras (1888) was among the first scientists to study DCI and proposed several procedures for gradual decompression. Katsaras detailed the pathogenesis and clinical forms of the disease, suggesting that divers should ascend slowly, in frequent intervals (1 min every 2 fathoms), to prevent DCI [13]. Around the same time, the Englishman Haldane and his colleagues were intensively studying divers’ physiology. They observed that a diver could emerge from a depth of up to 33 ft, regardless of the duration of stay, without developing symptoms. Thus, they concluded that the human body could tolerate a 2:1 pressure reduction without problems. Based on this, Haldane created a mathematical model for N2 dilution in the body, which could be used for depressurization processes (by stage depression). This gradual decompression approach is still being used worldwide. Notably, in 1908, Haldane published the first decompression tables, which have since been modified to cover even greater depths. He was also the first to use the term "half time" for tissue, after finding that body tissues absorb nitrogen to different degrees, depending on their vasculature and composition. The introduction of decompression tables enabled diving to progressively greater depths and the development of diving equipment [15].

The use of decompression tables

**Figure 1:**
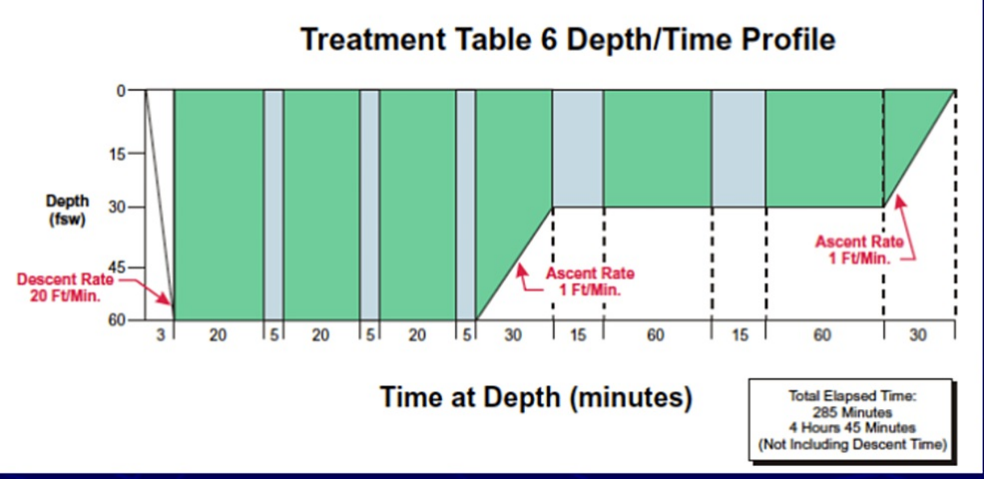
An example of a decompression table widely used by the United States Navy today. Feet sea water. Adapted from the United States Naval Sea Systems Command, US Navy Diving Manual, AquaPress, Washington, DC, 2008.

Initially, there was no consensus about DCI management. The first approach described recompression of divers at the depth of their dive. Another proposal included the diver's recompression at the depth where symptoms were relieved, while others suggested recompression at the depth of symptom relief plus an additional atmosphere. Similarly, the decompression process was also a debatable issue. The first attempt for unanimous and coordinated DCI guidelines was made by the US Navy in 1944-1945, resulting in the creation of the well-known hyperbaric treatment schedules or hyperbaric treatment tables. These tools received international recognition, offering a major advance compared to the previously established procedures. The basic principle of hyperbaric treatment tables involved the re-compression of the affected diver at the depth where symptoms retreated plus 1 to 6 atm. The decompression process, starting from the diver’s stay at the deepest point and occurring in frequent intervals, ranged from 6 to 38 hours. This prolonged period, despite not being eagerly accepted by divers, was the only alternative for DCI management. A few years later, in 1947, Edgar End began to manage real-life cases of decompression sickness. He argued that since the disease was associated with nitrogen, breathing air at high pressures increased the amount of nitrogen in the diver's tissues. The duration of treatment in his experiments lasted for 1-2 hours, followed by decompression. Notably, the results of his method were very encouraging. Unfortunately, in 1964, 20 years after the creation of the hyperbaric treatment tables, an increased incidence of treatment failure was observed. Indicatively, the failure rates in DCI treatment, in terms of neurological symptoms, reached 47%. This led Workman and Goodman to re-evaluate the use of oxygen at low pressures as a primary therapeutic measure. After three years of systematic research, in 1967, hyperbaric oxygen treatment tables, as well as tables for the treatment of cerebral air embolism, were finally published. The novel, more effective procedures that lasted significantly shorter (ranging from 135-285 minutes) replaced the previous ones and were soon globally accepted [16]. An example of a decompression table widely used today by the United States Navy is illustrated in Figure 1.

Building hyperbaric chambers

The progress in the field of mechanics has significantly contributed to the management of decompression illness (DCI). Despite the long history of Hyperbaric Medicine, the first hyperbaric chamber was constructed in 1662.

Henshaw (The First Hyperbaric Chamber)

In 1662, before the discovery of the oxygen method by Scheele, a British clergyman without any scientific background was associated with the first hyperbaric chamber. Henshaw increased the pressure in a sealed chamber using special valves, which he called "domicilium." The underlying rationale was simple: severely affected patients could benefit from increased air pressure, while those with chronic conditions could benefit from reduced air pressure [17].

Europe

About a century later, in 1782, the Dutch Academy of Sciences was assigned the task of designing a device to study the biological consequences of increased pressures, but no conclusive data emerged. A significant milestone in the history of hyperbaric medicine occurred in 1832 when Emile Tabarie, a physician in Montpellier, France, presented a study on the consequences of air pressure (2-4 atmosphere absolute (ATA)) to the French Academy of Scientists. Later, in 1837, another French clinician, Pravaz, built the largest hyperbaric chamber of that time, a room housing 12 people where pressurized air was pumped. This chamber was used to treat a variety of medical conditions, such as pulmonary diseases (e.g., tuberculosis, laryngitis), hearing loss, cholera, rickets and osteomalacia, menorrhagia, and conjunctivitis.

Over time and especially in the last few decades, the use of hyperbaric oxygen therapy has evolved. Of note, the perpetual efforts of the European Commission of Hyperbaric Medicine (ECHM) and the European Underwater and Baromedical Society (EUBS) have played a major role in the rapid development of hyperbaric oxygen therapy. These institutions are dedicated to the study and promotion of diving and hyperbaric medicine, serving as vital sources of medical information. They provide indications for hyperbaric oxygen therapy, therapeutic protocols, and set standards for technical procedures, equipment, training, and safety during treatment. Additionally, decompression tables are constantly under revision to minimize the risk of decompression sickness [18].

America

The first decompression chamber in North America was built in 1860 in Oshawa, Ontario, Canada. A year later, Corning Inc. constructed the first hyperbaric chamber in New York for the treatment of neurological disorders, among other diseases. Currently, in the United States, the steadily growing use of hyperbaric oxygen therapy resulted from the actions of the diving and hyperbaric medicine company, UHMS. UHMS was established in 1967 by medical practitioners and scientists as an international non-profit association. In October 1975, 50 leading hyperbaric investigators from different parts of the world developed the frame of research and indications for hyperbaric oxygen therapy [1] Today, this company employs approximately 2,000 medical, scientific, and nursing personnel in 50 countries. UHMS represents the primary source of information on hyperbaric and diving medicine worldwide in terms of: a) providing a scientific forum in basic and applied research for the treatment of patients with hyperbaric oxygen; b) promoting collaboration among physicians and other healthcare professionals (c) providing information and support regarding legislative and regulative changes; (d) developing and promoting educational activities and conferences that promote the scientific knowledge in diving and hyperbaric oxygen treatment; and (e) increasing the quality of treatment throughout the field of hyperbaric medicine by promoting high standards and widely accepted evidenced-based guidelines.

Greece

In Greece, hyperbaric oxygen therapy is closely linked with the local history of diving and the Hellenic Navy, which used to be the sole provider of decompression chambers and training for health professionals. The first decompression chamber, capable of accommodating only one individual, was installed on the deep-sea tugboat 'SOTIR' in 1957. Due to increasing demands, the Hellenic Navy General Staff acquired a multi-person decompression chamber, installed at the Naval Hospital of Piraeus in 1963. In 1970, another two-compartment chamber was deployed at the Nautical Hospital of Crete. In 1981, the chamber at the Navy Hospital of Piraeus was transferred to the Salamis Navy Hospital, where its scope gradually expanded to include diseases not exclusively related to diving, such as chronic osteomyelitis and diabetic ulcers. In April 1997, the decompression chamber was moved to the Athens Navy Hospital. Since then, the number of indications and consequently, the number of patients using hyperbaric oxygen therapy has increased further. Since 1994, the Hellenic Navy has established the medical specialty of Hyperbaric-diving medicine in Greece. Over the last three decades, interest in developing decompression chambers has grown, with many now existing in both public and private healthcare settings across Greece, such as in the "Vouvalio" Hospital of Kalymnos and the General Hospital "Agios Pavlos" in Thessaloniki [19].

The future of diving medicine and its prospects

Hyperbaric-diving medicine holds an important place in modern medical science. The course of its evolution over the centuries proves that humans not only have the innate need for continuous exploration of their potential, but also possess an unstoppable desire for understanding the unknown. The exploration of the seabed began when human beings abandoned their natural environment and felt ready to push their abilities to the limits. With the advent of technology enabling deeper explorations, a rapid development took place. During the centuries, any arising problem triggered further scientists’ curiosity in a quest to improve human health and quality of life. Various diseases, such as DCI and arterial gas embolism, are directly linked with Hyperbaric Medicine. Beyond these, hyperbaric medicine can be synergistically applied with traditional therapeutic methods, offering excellent outcomes in several other conditions, including gas gangrene, ulcer healing, spinal cord injuries, and osteomyelitis [20, 21]. Concurrently, although still under investigation, hyperbaric oxygen therapy shows promising potential for treating various medical disorders, such as inflammatory bowel disease, fractures with delayed healing, and severe retinal artery occlusion [22-27]. However, the adverse effects of hyperbaric oxygen therapy do exist and are the subject of ongoing research [28]. Before concluding, it is also important to mention the contribution of hyperbaric-diving medicine in areas not directly related to healthcare. First of all, it obviously holds an important role in the field of scientific diving (with the recent example of exploring the shipwreck of Antikythera for technical and scientific purposes). Moreover, Hyperbaric-Diving Medicine contributes to organizing military operations (i.e., escape from submarines and the activities of Underwater Demolition Unit). Finally, let's not underestimate the fact that diving is nowadays a recreational sport that attracts many people. Therefore, in terms of diving tourism, it can offer substantial support, ensuring both health and safety [29].

## Conclusions

Diving medicine is the field of medicine that serves the diagnosis, treatment, and precautions against illnesses that are related to diving activities, especially DCI. Although the history of diving and hyperbaric medicine is very long, the recent progress in the field of mechanics has offered great contribution and expanded our knowledge, capacity, and its indications. A conjunction of technology, clinical research, and genetics points in this direction, promising a bright future not only for healthcare professionals associated with this field but for society as a whole.
